# Impact of students assistance policies on quality of life and mental health

**DOI:** 10.3389/fpsyg.2023.1266366

**Published:** 2023-11-14

**Authors:** Lara dos Santos de Brito, Tiago Novaes Pereira, Emerson Roberto dos Santos, Thales Guardia de Barros, William Donegá Martinez, Loiane Letícia dos Santos, Vânia Maria Sabadoto Brienze, Alba Regina de Abreu Lima, Thaís Santana Gastardelo Bizotto, Júlio César André

**Affiliations:** Center for Studies and Development of Health Education – CEDES, Faculdade de Medicina de São José do Rio Preto, São José do Rio Preto, Brazil

**Keywords:** medical students, student assistance policies, quality of life, mental health, minor mental disorders

## Abstract

**Introduction:**

Student assistance policies in higher education, in their various modalities, seek to reduce the dropout of a new profile of students, non-traditional, with socioeconomic weaknesses, promoting quality of life (QoL) and mental health during the university journey. In this context, the Student Social Support Center (C.A.S.A) promotes assistance to students who need personal and/or economic support.

**Objectives:**

To evaluate the QoL and the presence of minor mental disorders (MMD) in students from the 1st to the 4th year of medicine at a public college in Brazil, comparing C.A.S.A beneficiaries and non-beneficiaries.

**Materials and methods:**

Cross-sectional study with 283 students. SRQ-20 and WHOQOL-BREF questionnaires were used, in addition to a questionnaire addressing sociodemographic data.

**Results:**

The general average of QoL was regular in the four evaluated domains (physical, psychological, social relationships, environment) and 55.5% of the students have evidence of MMD, in which the QoL scores are lower in all domains. The environment domain, which discusses socioeconomic conditions, has the worst score among C.A.S.A beneficiaries and the best among C.A.S.A non-beneficiaries.

**Discussion:**

The data corroborate the fragile situation of mental health and QoL of medical students. The student assistance modality of the analyzed program possibly presents vulnerabilities in its performance since the environment domain, the one with the lowest score among the program beneficiaries, precisely encompasses financial resources, access to goods and leisure and the individual’s physical environment.

## 1. Introduction

The World Health Organization (WHO) defines quality of life (QoL) as “the individual’s perception of their place in life, in the context of the culture and value systems in which they live and in relation to their goals, expectations, standards and concerns.” Its evaluation is made by the individual perception of satisfaction with life and with various aspects that compose it (Group W, 1994). Regarding medical students, their QoL and mental health were lower than the general population in several studies (Dyrbye et al., 2006; Gan and Hue, 2019; Maser et al., 2019; Quek et al., 2019; Solis and Lotufo-Neto, 2019; Miguel et al., 2021), including a systematic review and meta-analysis of 195 studies involving 129,123 medical students in 47 countries (Rotenstein et al., 2016). Several factors can contribute to this reality, such as strenuous workload (Damiano et al., 2021), sleep deprivation and other negative impacts on physical health (Hill et al., 2018), financial stress (Shao et al., 2020) and higher prevalence of mental disorders such as anxiety, depression and Burnout Syndrome (Moir et al., 2018). The estimated frequency of depression and its manifestations among medical students in systematic reviews and meta-analyses worldwide was 27.2%, while that of anxiety showed different percentages of prevalence depending on the place of study, ranging from 6.6 to 73% (Mirza et al., 2021). However, this prevalence in the world population, in 2019, was around 3.6% for depression and 3.9% for anxiety, much lower percentages when compared to medical students (World Health Organization (WHO), 2022).

Emphasizing the financial and socioeconomic issue, in low-income countries such as Yemen, worse QoL was evidenced, especially when analyzing psychological and environmental aspects, of those students with restriction in the continuous supply of water and difficulties in accessing electricity (Obad et al., 2021). In addition, there are aggravating factors among minority and vulnerable students, including ethnicity and economic history, with greater financial stress experienced by these groups (McMichael et al., 2022), which can also have consequences for QoL. Although these students are still underrepresented in medical schools (Baugh et al., 2019), it is important to highlight the change in the profile of the university student body currently underway, with a more significant portion of students identifying with these groups. In the United States, in 2016, 71% of higher education students had at least one characteristic of non-traditional students (Martinez et al., 2020), characterized as financially independent from parents, with one or more dependents, late entry into higher education, without a traditional high school diploma, in part-time courses, and individuals employed full-time while in school (Larin, 2018). In Latin America, this trend continues, with a greater intake of women, low-income people, older people and people of different ethnicities, other than Caucasians (Sandoval, 2022). Although these data show progress in terms of access to higher education for minority groups, this new demographic composition can bring new challenges, such as new sources of financial stress, which can result in unfavorable outcomes, from the academic to the personal scope.

A study in Australia pointed to financial stress as a factor that affects the well-being of medical students (Rogers et al., 2012), while in Canada, concern about finances is among the most common causes of stress among medical students (McLuckie et al., 2018). Financial stress is the cause of mental health deterioration, which results in poor academic performance, academic dishonesty, in addition to mental disorders such as anxiety, depression and Burnout Syndrome (Pokhrel et al., 2020). Furthermore, food insecurity arising from worse socioeconomic conditions is associated with worse college grades (Martinez et al., 2018).

Given this context, it is important to create conditions for the permanence of these more vulnerable students in college. One possibility is financial aid, offered today in different ways with different requirements. One of these categories, performance-based financing, did not bring major advances to higher education students when analyzing graduation and retention rates, in addition to making colleges more selective in choosing their students, excluding those with greater vulnerabilities and minor chances of completing the course (Kelchen, 2017). Another category is providing loans, which create debt for students. A systematic review points to worse academic performance and worse mental health of students with debts, with greater impairment of rural populations, who are part of the most vulnerable group of students (Pisaniello et al., 2019). Another possibility is scholarships without the need for a counterpart, taking into account the socioeconomic conditions of the students.

The National Scholarship Program (NSP), a funding program for low-income students in England, is similar to this model of financial aid without counterparts (National Scholarship Programme, 2011). Despite the program’s impacts still requiring further evaluation, after its implementation there was a reduction from 9.1 to 5.7% in the higher education discontinuity rate from 2003/4 to 2012/13 (European Commission et al., 2015). Furthermore, most institutions stated that the NSP helped the retention rate of disadvantaged students, and 40% of beneficiaries stated that the program had a significant influence on the process of choosing the institution. In addition, students made positive remarks about the program, noting an improvement in their ability to focus on their studies and the possibility of purchasing necessary materials, such as text books (Bowes et al., 2016).

The assessment of QoL and mental health in medical students are widely reported in the literature, however little is known about these parameters in students benefiting from student stay policies compared to the rest of the student body, as a way of assessing their effectiveness and ability to make college an equitable environment.

The study aimed to evaluate the influence of a program that provides assistance to medical students on the QoL and Mental Health of those attended by it. The program in question is aimed at students who present, in their social expression, a state of imbalance that requires guidance and support in the personal, academic and/or economic fields called C.A.S.A (Student Social Support Center), and is carried out through financial support in the form of a cash grant and access to free food at a public medical school in Brazil. The first aspect assessed was QoL, which discusses the psychological, physical, environmental and social relations domains. As a complement to the study, the risk of minor mental disorders (MMD) in students was analyzed, defined as a set of depressive, anxious and psychosomatic symptoms that do not meet the formal criteria for mental disorders defined by the International Classification of Diseases – ICD (Goldberg and Huxley, 1992). The assessment of MMD is interesting, given the direct relationship between mental health and socioeconomic status, in addition to the alarming indicators about the mental health situation of medical students, mentioned above. In short, this study aimed to evaluate QoL indicators and the presence of MMD in students from the 1st to the 4th year of medicine, comparing students who benefited from the C.A.S.A and students who did not. The purpose of this evaluation is to provide support for possible discussions about greater effectiveness in the services offered by this program, as well as others with a similar model.

The study’s objective is to assess how the C.A.S.A program impacts the QoL and the presence of MMD in medical students who receive benefits from the program, and to compare these indicators with students who do not receive such benefits. The study is important to understand how the program influences the socioeconomic disparity between these two groups and whether it is capable of reducing this disparity in order to ensure the retention of students from lower socioeconomic backgrounds in the course. Furthermore, this study is of significance given the limited available literature on the subject and the fact that retaining underprivileged students through student assistance policies is a means to enhance diversity in the academic environment and expand study and employment opportunities for this population.

## 2. Materials and methods

Cross sectional study with analytical components, developed in 2018, after approval by the Research Ethics Committee of the Medicine College of São José do Rio Preto (CEP/FAMERP) under number 2.666.947, of May 22, 2018.

### 2.1. Sample

The sample consisted of undergraduate students in Medicine at FAMERP from the 1st to the 4th year, approved, regularly enrolled and attending, in 2018, out of a total of 320 students from the 1st to the 4th year. For comparative data analysis, the sample was divided into 2 groups: C.A.S.A students and non-beneficiary students.

The selection process to become a C.A.S.A beneficiary assesses the socioeconomic condition of the students, especially in terms of family income, who voluntarily signed up for the program, to ensure access for those in the worst socioeconomic condition, given the limited number of vacancies. When this work was being carried out, C.A.S.A offered assistance in the form of cash grants deposited in the student’s personal bank account (stay allowance), access to free food from the cafeteria of the hospital linked to the institution and a small portion of grants with the provision of 20-h weekly service to the faculty (student aid).

### 2.2. Data collection

The survey took place on the college campus, and a self-reported questionnaire in physical form was administered. The scheduling of test times (during college activities) was done in advance by the researchers to maximize student participation. The research was presented to the participants before the start of the selected activity for test administration (most of which were in-person institutional assessments and tasks), and they were given the option to complete the research form at the end of the activity. The data were then entered into the Microsoft Excel program for further analysis. The questionnaire consisted of two widely used self-administered instruments, translated into Portuguese and validated, for screening and detecting possible MMD and QoL indicators. These instruments were:

#### 2.2.1. Self-report questionnaire-20

This questionnaire comprises 20 questions addressing physical and emotional health-related matters. It is a validated instrument designed for the Brazilian population and has been translated into Portuguese. In the context of the Brazilian population, a cutoff score of 6/7 is defined on a scale ranging from 0 to 20. This cutoff is determined by summing the affirmative responses to the questionnaire’s questions. Scores above the cutoff are indicative of a potential risk for a MMD (Oliveira Bernardes Santos et al., 2010).

#### 2.2.2. World Health Organization quality of life-BREF

This questionnaire comprised 24 questions covering four domains pertinent to QoL, which are psychological, physical, environmental, and social relationships. Additionally, it includes two distinct questions concerning the individual’s self-assessment of their QoL and health. All questions are rated on a scale ranging from 1 to 5, with higher scores indicating more favorable parameters for the individual. To assess the domains, the responses from each question are aggregated and averaged, and this can be interpreted on a scale from 1 to 5, with a higher score signifying a more favorable domain parameter. In terms of perceived QoL, it can range from ‘very poor’ to ‘good,’ while the perception of health can vary from ‘very dissatisfied’ to ‘very satisfied.’ This instrument is also validated and translated into Portuguese (Fleck et al., 2000).

As a socioeconomic indicator, an original questionnaire developed by the researchers was used. This questionnaire addressed key aspects potentially influencing a student’s QoL and mental health, including income, ethnicity, gender, access to psychological or psychiatric treatment, and eligibility for C.A.S.A scholarships. Importantly, the questionnaire was designed to ensure the anonymity of the students and did not contain any identifying information.

### 2.3. Data analysis

Exploratory data analysis included descriptive statistics, mean, median, standard deviation, minimum and maximum value for numeric variables and number and proportion for categorical variables. For the analysis of the behavior of continuous variables, descriptive statistics, histogram and boxplot graphs and the specific test for the theoretical assumption of Shapiro–Wilk normality were considered (Conover, 1999).

Comparison of categorical variables between groups was performed using Pearson’s Chi-square test or Fisher’s exact test, when appropriate. It should be noted that Pearson’s Chi-square test does not assume the ideal sample size for its application, it is only recommended that the two variables be nominal categorical, the samples must be independent and the observations must be summarized in frequencies or counts (Siegel and Castellan, 2006).

Comparison of numerical variables with normal distribution between two groups was performed using Student’s *t*-test. However, considering the low number of cases in the group “C.A.S.A beneficiaries,” in spite of being a characteristic of the study population, on the analysis based in resampling with replacement called Bootstrap was performed. The Bootstrap procedure is a resampling technique widely used in different statistical situations, mainly for evaluating estimates of parameters produced by statistics, distribution errors, among other situations (Tibshirani and Efron, 1993). In this study, the Bootstrap technique was used to evaluate the properties of parameter estimates, standard error of the distribution and the convergence of the probability of significance of the analyzed variables. Statistical analysis was performed using the software IBM-SPSS Statistics version 28 (Software SPSS, 2023).

## 3. Results

Through the application of the sociodemographic questionnaire, it is possible to characterize the students. Demographic data indicate that 52.6% of the students were male, and 74.7% were white, followed by brown (11%) and Asian (9.9%). Black individuals accounted for only 1.8% of the students. The majority of students come from families with monthly earnings above 3 minimum wages, accounting for 89%, indicating that they are mostly from the upper-middle class. Most do not live with their families (90.1%) and have infrequent visits home. 68.8% of students take more than 15 days to visit their families.

Regarding students benefiting from C.A.S.A, they make up 10.2% of the total number of students (28 students). Among the assistance modalities received, the most prevalent is the provision of free meals, with 53.6% of beneficiaries receiving only this assistance, without cash grants.

Regarding mental health-related data, considering the total group of students, 45.3% of students have a history of psychiatric and/or psychological treatment. Analyzing the data obtained by the SRQ-20, considering the total group of students, we find a risk of MMD in 55.2% of students, while suicidal ideation, one of the specific questions on the questionnaire, was positive for 8.9% of students.

Table 1 presents some demographic data for the total group of students, as well as a comparison of these data between C.A.S.A beneficiaries and non-beneficiaries. The comparative analysis between students with and without the C.A.S.A benefit showed a significant difference in terms of family income, with beneficiary students presenting lower income than non-beneficiaries. There were no differences in other sociodemographic aspects. Table 1 also presents the risk of MMD under the same comparison, also with no significant difference between the two groups.

**Table 1:** Demographic and comparative analysis of students included in the study, comparing beneficiary and not beneficiary of C.A.S.A.

Characteristic	Total (*n* = 283)	Beneficiary of C.A.S.A (*n* = 28)	Not beneficiary of C.A.S.A (*n* = 248)	Value of *p*^3^
**Gender, *n* (%)**				
Masculine	144/274 (52.6)	18 (64.3)	126 (51.6)	0.204
Feminine	128/274 (46.7)	10 (35.7)	118 (48.4)	
Not declared	2/274 (0.7)	-	2 (0.8)	
**Color, *n* (%)**				
White	204/273 (74.7)	19 (70.4)	185(75.2)	0.583
Non-white/not declared	69/273 (25.3)	8 (29.6)	61 (24,8)	
**Income, *n* (%)**				
Up to 3 wages	30/273 (11.0)	10 (37.5)	20 (8.2)	
More than 3 wages	243/273 (89.0)	18 (64.3)	225 (91.8)	**<0.001**
**Lives with family, *n* (%)**				
Yes	27/274 (9.9)	2 (7.7)	25 (10.1)	1.000
No	247/274 (90.1)	24 (92.3)	223 (89.9)	
**Return home, *n* (%)**				
Frequent (every 15 days)	77/247 (31.2)	7 (28.0)	70 (31.5)	0.718
Infrequent^1^	170/247 (68.8)	18 (72.0)	152 (68.5)	
**Psychiatric treatment, *n* (%)**				
Yes	123/271 (45,3)	11 (42.3)	112 (45.7)	0.740
No	148/271 (54,7)	15 (57.7)	133 (54.3)	
**Risk of minor mental disorders, *n* (%)** ^2^				
Yes	153/274 (55,8)	16 (61.5)	137 (55.2)	0.538
No	121/274 (44,1)	10 (38.5)	111 (44.8)	

Categorical variables described in number (percentage). ^1^Every 1–2 months, or only on long holidays/vacations. C.A.S.A - Student Social Support Center. ^2^Data extracted from the application of the SRQ-20 questionnaire, considering a cutoff score of 7, valid for the Brazilian population. ^3^*p* values are based on Pearson’s Chi-square test or Fisher’s exact test, when appropriate, between the two groups (beneficiary and not beneficiary of C.A.S.A). The bold values represent the best statistically significant results.

Table 2 provides a more in-depth analysis of the data obtained by the SRQ-20 when comparing individuals with and without the risk of MMD in relation to their sociodemographic characteristics, presenting some relevant results. Firstly, there was a trend for a lower proportion of students who frequently return home among students with possible mental disorders (*p* = 0.051). Suicidal ideation was present in 16 and 0% of students with and without the risk of MMD, respectively (*p* < 0.001), while the frequency of psychiatric or psychological treatment was also statistically different, being higher in students at risk for MMD (*p* = 0.001). There was also a significant difference between genders in the presence of MMD, with a higher prevalence in females (*p* < 0.001).

**Table 2:** Comparative analysis of demographic and clinical data among students with and without risk for minor mental disorders.

Risk of minor mental disorders			
Characteristic	Yes (*n* = 153)	No (*n* = 121)	Value of *p*^2^
**Gender, *n* (%)**			
Masculine	65 (42.8)	79 (65.8)	**<0**.**001**
Feminine	87 (57.2)	41 (34.2)	
**Color, *n* (%)**			
White	111 (72.5)	93 (77.5)	0.350
Non-white	42 (27.5)	27 (22.5)	
**Income, *n* (%)**			
Up to 3 wages	16 (10.5)	14 (11.6)	
More than 3 wages	136 (89.5)	107 (88.4)	0.784
**Lives with family, *n* (%)**			
Yes	18 (11.8)	9 (7.4)	0.233
No	135 (88.2)	112 (92.6)	
**Return home, *n* (%)**			
Frequent (every 15 days)	35 (25.9)	42 (37.5)	**0**.**051**
Infrequent^1^	100 (74.1)	70 (62.5)	
**Psychiatric treatment, *n* (%)**			
Yes	82 (54.3)	41 (34.2)	**0**.**001**
No	69 (45.7)	79 (65.8)	
**Suicidal ideation, *n* (%)**			
Yes	25 (16.0)	0 (0)	**<0**.**001**
No	131 (84.0)	125 (100.0)	

Categorical variables described in number (percentage). Data extracted from the administration of the SQR-20 questionnaire. ^1^Every 1–2 months, or only on long holidays/vacations. ^2^*p* values are based on Pearson’s Chi-square test or Fisher’s exact test, when appropriate, between the two groups (Yes and No for risk of minor mental disorders). The bold values represent the best statistically significant results.

**Figure 1:**
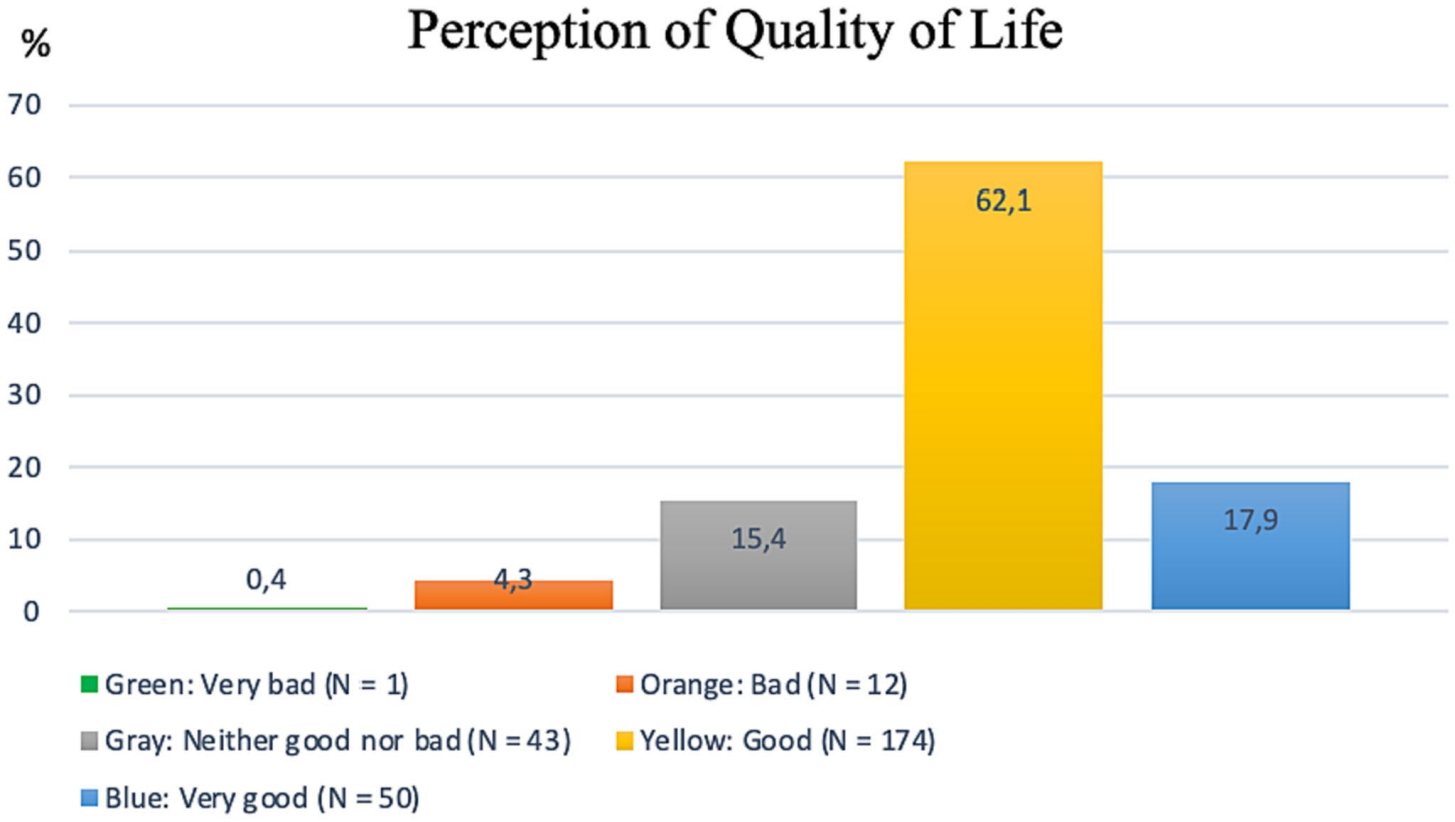
Perception of quality of life, according to WHOQOL-BREF. Data available on 280 students.

**Figure 2:**
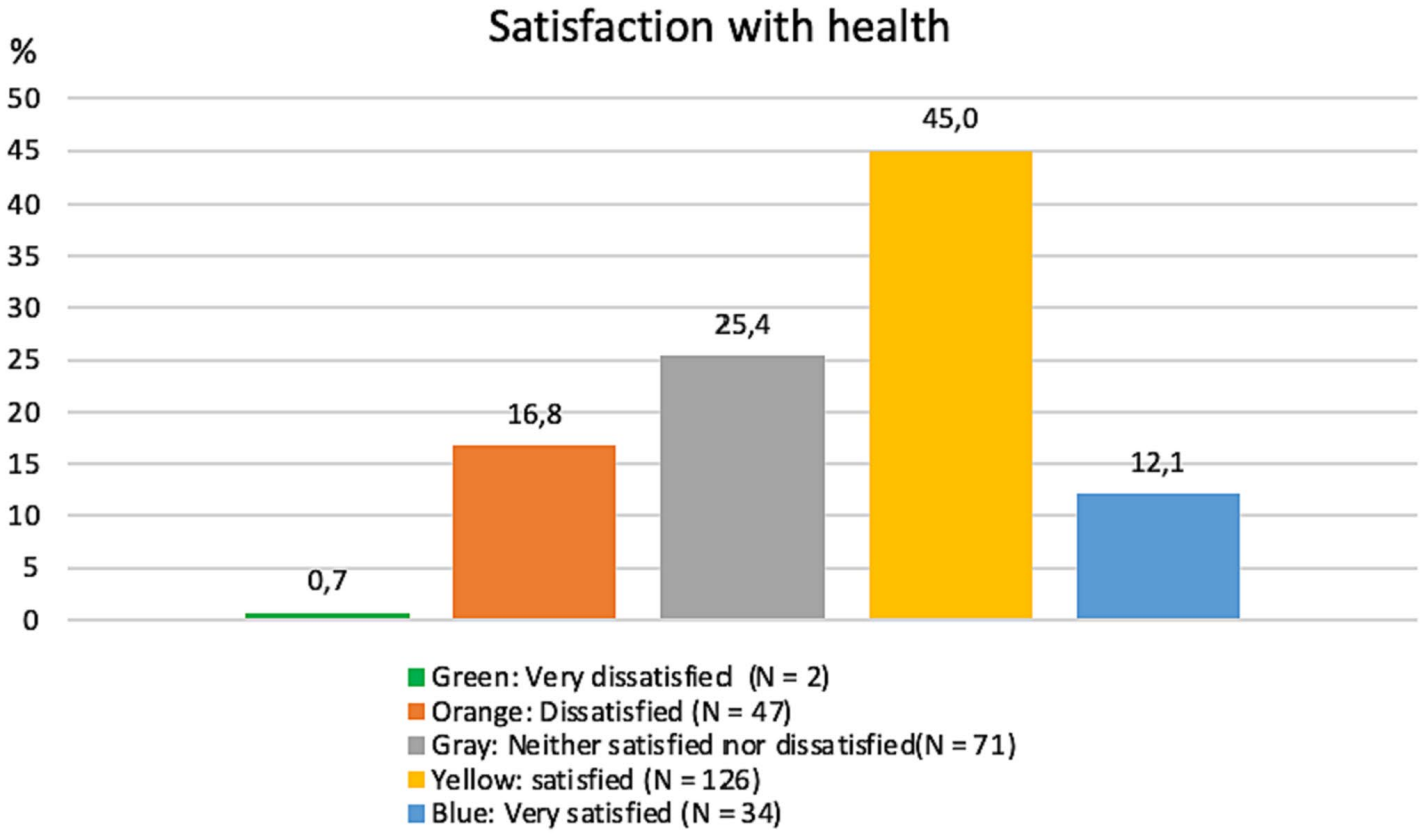
Satisfaction with health, according to WHOQOL-BREF. Data available on 280 students.

Regarding the analysis of the WHOQOL-BREF, first, there is an evaluation of all students concerning the first two questions of the questionnaire in Figures 1, 2. Figure 1 shows the individual’s assessment of their QoL, referring to the first question of the WHOQOL-BREF questionnaire, with a prevailing positive perception (62.1%), indicating that most students scored 4 on the question, on a scale of 1–5. Figure 2 evaluates students’ individual satisfaction with health, referring to the second question of the WHOQOL-BREF, showing that the majority of students (45%) are satisfied with their own health.

Continuing with the analysis of WHOQOL-BREF data, Table 3 brings a comparative analysis of the QoL between C.A.S.A beneficiaries and non-beneficiaries, as well as the assessment of the QoL for all students, including the four domains explored by the questionnaire. The perception of QoL, satisfaction with health among students, and assessment of the four domains covered by the instrument were considered at a regular level (between 3 and 4 on a scale of 1–5). As for the QoL indicators, there was a decline in QoL perception scores and the environment domain for students with C.A.S.A benefits.

**Table 3:** Comparative analysis of quality of life among students: beneficiaries versus non-beneficiaries of C.A.S.A.

	Total (*n* = 283)	Beneficiary of C.A.S.A (*n* = 28)	Not beneficiary of C.A.S.A (*n* = 248)	Value of *p*^2^
Perception of quality of life (mean ± SD)^1^	3.93 ± 0.73	3.68 ± 0.72	3.97 ± 0.70	0.038
Satisfaction with health (mean ± SD)^1^	3.51 ± 0.94	3.43 ± 0.92	3.52 ± 0.92	0.614
**Domain**				
Physical (mean ± SD)	3.57 ± 0.58	3.60 ± 0.59	3.57 ± 0.57	0.762
Psychological (mean ± SD)	3.50 ± 0.58	3.49 ± 0.46	3.50 ± 0.58	0.898
Social relationships (mean ± SD)	3.62 ± 0.75	3.59 ± 0.64	3.61 ± 0.75	0.906
Environment (mean ± SD)	3.73 ± 0.53	3.49 ± 0.53	3.76 ± 0.52	**0.015**

Numeric variables described as mean ± standard deviation. Data extracted from the administration of the WHOQOL-BREF questionnaire, considering a rating scale from 1 to 5, with higher scores indicating closer proximity to 5 for improved parameters. ^1^Data available for 28 and 245 students with and without C.A.S.A benefit, respectively. ^2^*p* values are based on Student’s *t*-test, between the two groups (beneficiary and not beneficiary of C.A.S.A). The bold values represent the best statistically significant results.

Finally, in Table 4, there is a comparative analysis of the QoL, according to the WHOQOL-BREF, between students with and without the risk of MMD. All QoL indicators (perception, satisfaction with health, and domains) had lower scores among students at risk of MMD when compared to those without the risk.

**Table 4:** Comparative analysis of quality of life, according to the WHOQOL-BREF, among students with and without minor mental disorders.

Risk of minor mental disorders			
	Yes (*n* = 156)	No (*n* = 125)	Value of *p*^2^
Perception of quality of life (mean ± SD)^1^	3.71 ± 0.74	4.20 ± 0.62	**<0.001**
Satisfaction with health (mean ± SD)^1^	3.15 ± 0.89	3.97 ± 0.79	**<0.001**
**Domain**			
Physical (mean ± SD)	3.33 ± 0.52	3.87 ± 0.49	**<0.001**
Psychological (mean ± SD)	3.24 ± 0.53	3.87 ± 0.49	**<0.001**
Social relationships (mean ± SD)	3.44 ± 0.76	3.84 ± 0.67	**<0.001**
Environment (mean ± SD)	3.57 ± 0.51	3.93 ± 0.50	**<0.001**

Numeric variables described as mean ± standard deviation. ^1^Data available for 124 and 156 students without and with minor mental disorders, respectively. ^2^*p* values are based on Student’s *t*-test, between the two groups (Yes and No for risk of minor mental disorders). The bold values represent the best statistically significant results.

## 4. Discussion

With regard to comparative data between C.A.S.A beneficiary and non-beneficiary students, a significant difference in income was demonstrated, which corroborates the fairness of the selection process for obtaining the benefit. There was no significant difference in the risk of MMD between the two groups, possibly due to the multifactorial nature of the medical student’s psychological suffering, which is not limited to the financial issue, as previously discussed. However, regarding QoL, it is possible to differentiate between the two groups.

The global assessment of QoL, considering the perception of QoL, satisfaction with health and the four domains analyzed by the WHOQOL-BREF, point to regular QoL, with scores between 3 and 3.9. When analyzing the domains comparing C.A.S.A beneficiary and non-beneficiary students, it is interesting to note that the first group has a worse perception of QoL and the lowest scoring domain is the Environment domain, while among non-beneficiary students this same domain has the highest score. The domain of the environment discusses exactly the financial resources, access to goods and leisure and the physical environment of the individual. Certainly, access to financial resources, as discussed within the environment domain, holds substantial importance within the context of medical school. Upon analyzing the socioeconomic profiles of students, especially those from Brazil, it is possible to observe a prevalence of higher social standings, even upon their entry into college (Dhalla et al., 2002; Fiorotti et al., 2010; Rego et al., 2018; Souza et al., 2020).

Although these results in relation to the environment domain are understandable given the worst socioeconomic conditions of students benefiting from C.A.S.A and the fact that non-beneficiary students probably would not need C.A.S.A support in the environmental domain due to their better socioeconomic condition, they may also be evidence of an attempt to remediate the failure of student assistance policies to guarantee equity between the two groups, precisely the objective from the program, mentioned in the institution’s bylaws (Faculdade de Medicina de São José do Rio Preto - FAMERP, 2001). The causes of this failure could be numerous, such as the type of benefit offered (mainly free food), the sufficiency of the amount offered in cash and the absence of other material resources to complement the benefit, such as free institutional transportation and student housing.

In addition to the data related to the central research question, other important data were analyzed, particularly regarding the characterization of the students. The prevalence of male students (52.6%) is in line with data from the student population in national medicine, composed in 2020 by 53.4% men (Scheffer et al., 2020). However, this percentage is close to the situation of equality between the number of male and female physicians, in line with the worldwide trend towards the feminization of medicine (Baig, 2020).

The non-absolute predominance of white students (74.7%) also reflects the national medical profile. In 2019, among the graduates of the medical course in Brazil, 67.1% declared themselves white (Scheffer et al., 2020). Furthermore, 89% of students earn more than three minimum wages. Considering that, in Brazil, 70% of the population earns up to two minimum wages (Instituto Brasileiro de Geografia e Estatística (IBGE), 2021), the high economic profile of higher education in Medicine in the country is evident.

Regarding mental health issues, the results show a risk of MMD in 55.5% of the total number of students. The result is similar to data from other medical schools, which show percentages from 60.5 to 29.6% (Conceição et al., 2019; Melado et al., 2019; Prata et al., 2021; Pereira et al., 2022). Despite the variable percentages, systematic reviews point to greater fragility in the mental health of medical students compared to the general population (Oliveira and Araujo, 2019). The rate of suicidal ideation, of 8.9%, is also in line with the literature, since the cumulative prevalence of suicidal ideation among medical students was 11.1%, based on a meta-analysis with students in 43 countries (Rotenstein et al., 2016). When analyzing the risk of MMD related to sociodemographic data, a significantly higher risk was found among women. In fact, the WHO points to a higher recurrence of mental disorders in women worldwide (World Health Organization (WHO), 2022). Several causes of this pattern are pointed out, especially the cultural influence of patriarchy that dictates taking care of one’s own health as intrinsic to the female gender, making it difficult to diagnose these disorders among men (Cardoso et al., 2022). In this same analysis, a trend towards a lower proportion of students returning home frequently was observed among students with MMD. Distance from the family nucleus can be a risk factor to worsen the student’s psychosocial condition (Silva et al., 2020).

Finally, the presence of lower scores in all QoL indices among students at risk for MMD, when compared to other students, is a strong indication of the multifactorial nature of the development of MMD. The WHO points to the multisectoral characteristic of mental health determinants and the need for an approach in several spheres for its care (World Health Organization (WHO), 2022). In the case of support for vulnerable medical students, the approach must also be multisectoral, represented by different policies that act on transportation, housing, leisure, community, among other factors of the student’s QoL and mental health, in addition to the financial benefit.

### 4.1. Limitations

There are some limitations to this study that should be acknowledged. Firstly, it was a cross-sectional study with data collected at a single time point. Therefore, to better assess the impact of student assistance policies, longitudinal tracking would be important. Secondly, C.A.S.A offers more than one form of assistance to students, making it challenging to determine which form (financial aid, free meals, student housing, among others) would be the most effective for retaining students with socioeconomic vulnerabilities in college. In this regard, conducting additional studies to evaluate each individual modality would be necessary. Lastly, there are potential biases and confounders when attributing QoL and MMD to C.A.S.A. It’s important to note that the medical course itself has unique characteristics, including an extensive workload, exposure to illness and death, a hidden curriculum, and more, which can potentially influence the mental health and QoL of medical students.

### 4.2. Final considerations

The present study sought to evaluate a model of student care for vulnerable students in relation to QoL and risk of MMD. Despite the groups having different sample sizes, which is a characteristic of the study population, the properties of the estimates of the parameters of the analyzed variables resulting from the application of the statistical test were adequate and consistent. This fact was evidenced by applying the Bootstrap technique with the purpose of evaluating the parameter estimates, standard error of the distribution and the convergence of the probability of significance.

Student assistance policies can play a decisive role in student education, especially non-traditional ones, by preventing dropouts and contributing to their academic and social development within the faculty. The present study seeks to evaluate a care model and support discussions on possible service improvements, in addition to complementing the already worrying data about the mental health of medicine students.

The mental impairment of medical students is a multifactorial process, influenced not only by socioeconomic variables, but by other determinants of QoL, such as health, social relationships and the environment. However, the structure of the current model of assistance and student permanence does not take these nuances into account, and is therefore insufficient in dealing with the high rates of MMD and suicidal ideation, as well as promoting the general well-being of the student. The worrying panorama of medical students’ mental health is not only a consequence of stressors and challenges inherent to the course, but also of a systemic failure on the part of managers and support systems to promote effective interventions that are not restricted to just one facet of the problem, but truly holistic in their planning and execution.

## Data availability statement

The original contributions presented in the study are included in the article/supplementary material, further inquiries can be directed to the corresponding author.

## Ethics statement

The studies involving humans were approved by Research Ethics Committee of the Medicine College of São José do Rio Preto (CEP/FAMERP). The studies were conducted in accordance with the local legislation and institutional requirements. The participants provided their written informed consent to participate in this study.

## Author contributions

LB: Conceptualization, Investigation, Writing – original draft, Writing – review & editing. TP: Conceptualization, Investigation, Writing – original draft, Writing – review & editing. ES: Writing – review & editing. TBa: Writing – review & editing. WM: Writing – review & editing. LS: Writing – review & editing. VB: Formal analysis, Writing – review & editing. AL: Writing – review & editing. TBi: Writing – review & editing. JA: Formal analysis, Methodology, Project administration, Supervision, Writing – review & editing.
